# Root repair after damage due to screw insertion for orthodontic miniplate placement

**DOI:** 10.4317/jced.56472

**Published:** 2019-12-01

**Authors:** Marie A. Cornelis, Michele Tepedino, Paolo M. Cattaneo, Catherine Nyssen-Behets

**Affiliations:** 1 Associate Professor,Section of Orthodontics, Department of Dentistry and Oral Health, Aarhus University, Faculty of HEALTH, Vennelyst Boulevard 9, 8000 Aarhus, Denmark; 2 Associate lecturer, Department of Biotechnological and Applied Clinical Sciences, University of L’Aquila, Via Vetoio 2, 67100 L’Aquila, Italy; 3 Associate Professor, Section of Orthodontics, Department of Dentistry and Oral Health, Aarhus University, Faculty of HEALTH, Vennelyst Boulevard 9, 8000 Aarhus, Denmark; 4 Professor, Pôle de Morphologie, Institut de Recherche expérimentale et clinique, Université catholique de Louvain, Avenue Mounier 52 bte B1.52.04, 1200 Brussels, Belgium

## Abstract

**Background:**

The aim of this investigation was to describe the healing reactions following root damage caused by placement of a miniplate anchorage system.

**Material and Methods:**

In 4 beagle dogs, 4 titanium miniplates (2 self-tapping screws per miniplate) were placed in each maxilla, after drilling of pilot-holes. Six fixation screws were unintentionally inserted damaging the root of maxillary canines. Two weeks later, half of the miniplates were loaded with a coil spring. Two dogs were euthanized 7 weeks after placement of the miniplates, while the remaining two after 29 weeks. Histological sections were prepared, microradiographed, observed under U.V. light, then stained and analysed under ordinary light.

**Results:**

Four screws caused direct root damage; one was damaged during the drilling process; one caused damage to the periodontal ligament only. Among these 6 screws, 2 were mobile and 4 were stable at sacrifice. Limited root damage showed some repair after 29 weeks, consisting in a thick layer of mineralized cementum including anchoring periodontal fibres. Tissue repair was not related to screw stability or loading status.

**Conclusions:**

Limited root damage has shown potential to heal, while extensive root damage has not. Precise position of insertion of the miniplates is thus of utmost importance.

** Key words:**Temporary anchorage devices, animal studies, root resorption.

## Introduction

Miniplates and miniscrews have revolutionized orthodontic anchorage concepts (1,2). However, while their advantages are now well-described, time has come for evaluating their side effects. The risk of damaging the roots during placement of those temporary skeletal anchorage devices cannot remain underestimated (3-9). Tissue reactions following root lesions due to drilling and screw insertion for orthodontic miniplates placement have not been reported. The extension and severity of root damage might play an important role for the safe healing of the injuries sustained. Therefore, we report here retrospectively root damage which occurred during an animal investigation focusing on a miniplate anchorage system (10).

## Material and Methods

**Figure 1:**
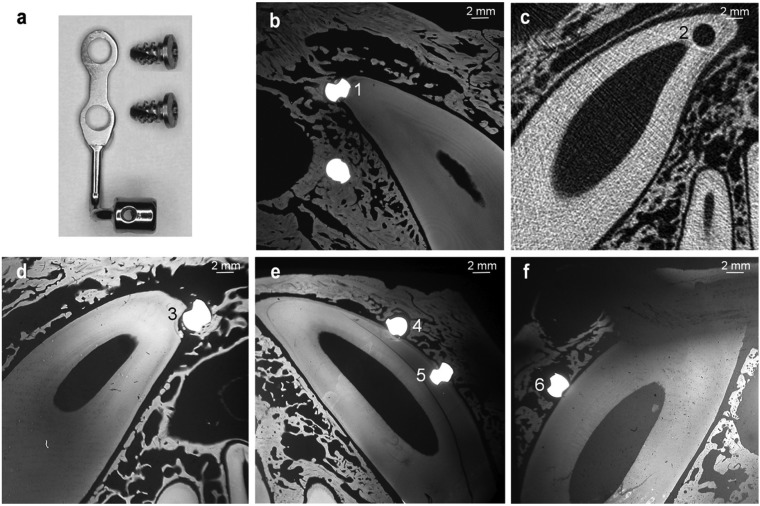
A) Bollard miniplate and fixation screws. B) Microradiograph showing both screws of miniplate I in dog A. C) CT scan depicting the hole drilled for miniplate II in dog B. D) Microradiographs showing the upper screw of miniplate III in dog C. E) screws of miniplate IV. F) the lower screw of miniplate V in dog D.

In four 1-year-old male beagle dogs, 16 titanium orthodontic miniplates (Surgi-Tec, Belgium) (Fig. 1a), were implanted between the maxillary dental roots (two miniplates per quadrant) each with two 5 mm-long, 2.3 mm-diameter TiAl6V4 self-tapping screws (Surgi-Tec). These dogs were part of a previously described larger sample (10 dogs & 40 maxillary miniplates) used to analyze the bone healing reactions after miniplates placement (10). The protocol was approved by the Animal Experimentation Ethics Committee of the Université catholique de Louvain.

For implantation, mucoperiosteal flaps were elevated, and pilot-holes were drilled with a 1.6 mm diameter bur under saline irrigation. After screwing, the incisions were sutured. Then, the dogs were given antibiotics and anti-inflammatory drugs for 5 days. The root or the PDL of the upper canine was hit during the drilling process of five miniplates in total. Three dogs (A, B, C) had one miniplate involving root contact, in either the right or left anterior maxilla (Fig. 1b-d), while dog D had 2 miniplates involving root damage: one in the left and one in the right anterior maxilla (Fig. 1e-f) ( Table 1). Two weeks later, half of the miniplates were loaded with a coil spring (125 g), while the other miniplates were left unloaded. Among the 5 miniplates involving roots damage, only miniplates I and V (Fig. 1b,f) were loaded ( Table 1).

**Table 1:**
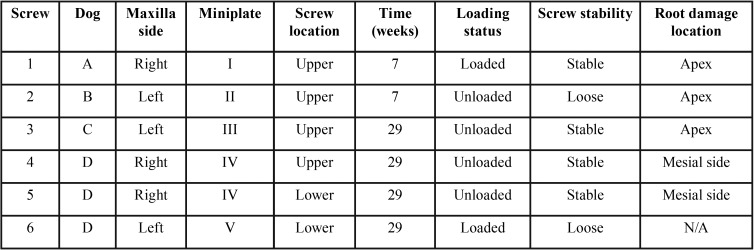
Distribution of screws according to dog, maxilla side, miniplate, upper or lower location within the miniplate, time to sacrifice, loading status, screw stability at sacrifice and location of root damage.

Two dogs were euthanatized 7 weeks after implantation (dogs A, B) and the remaining two after 29 weeks (dogs C, D). Stability of the miniplates was checked at sacrifice. Intravital bone-labeling fluorochromes were injected at placement (calcein green, intraperitoneally, 20 mg/kg) and 30 minutes before sacrifice (alizarin complexone, intravenously, 30 mg/kg).

Tissue blocks from the jaws including the miniplates were dissected, fixed and embedded in methylmethacrylate. Sagittal Computed Tomography (CT) sections were obtained with a peripheral Quantitative Computed Tomography (pQCT) Research SA+ (Stratec, Germany). Subsequently, the blocks were cut into 80-µm-thick, undecalcified sections, perpendicular to the screw axis. These sections were placed in contact with a fine grain emulsion and exposed to long wavelength X radiations produced by a Machlett tube (14 kV – 15 mA). The films were developed and mounted like histological samples. The sections were observed under U.V. light. The sections were then superficially stained with 1% fuchsin alcoholic solution or with a 1% aqueous solution of methylene blue buffered with potassium biphthalate at pH 4.8 and were observed under ordinary light.

## Results

Four screws directly damaged the upper canine roots (Fig. 1b,d,e - screws 1 and 3-5): the upper screw of miniplates I and III and both screws of miniplate IV. The upper screw of miniplate II did not hit the root itself, but the hole was drilled deeper than what required by the actual screw length, damaging the root (Fig. 1c - screw 2). Finally, the lower screw (Fig. 1f - screw 6) of miniplate V went into the PDL without reaching the root. Screws 1, 2 and 3 (Fig. 1b,c,d) injured the root apex while the remaining screws hit the mesial side of the canine root (Fig. 1e - screws 4 and 5) or its PDL (Fig. 1f - screw 6).

Among these 6 screws, 2 were loose and 4 were stable at sacrifice ( Table 1). This distribution was not related to the type of root damage or the loading status of the miniplates.

**Figure 2:**
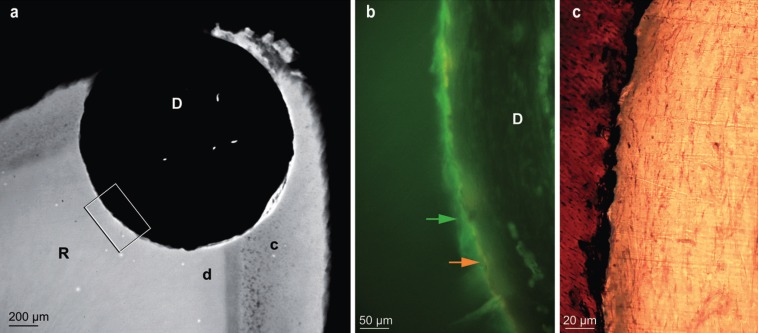
A) Microradiograph showing the hole drilled for screw 2 (7 weeks), damaging the root at the apex level. B) Enlargement of framed area in Fig 2a, examined in the section under UV light, showing the juxtaposition of calcein green (green arrow) and alizarin complexone (orange arrow) labelling. C) Enlargement of the area indicated by the arrows in Fig 2b, observed in the stained section. D: defect - R: root - c: cementum - d: dentin.

Microradiography as well as fluorescence microscopy did not show any root repair after 7 weeks (screws 1, 2): neither resorption nor new tissue was visible and alizarin complexone, injected just before sacrifice, appeared immediately adjacent to calcein green’s labelling, injected at screw insertion, attesting the absence of new dental tissue apposition (Fig. 2a,b). However, presence of fluorescent markers attested normality of calcium exchanges along the mineralized surfaces. The stained sections showed dentin in contact with loose connective tissue. No sign of inflammation was observed either in the PDL or in the pulp (Fig. 2c).

**Figure 3:**
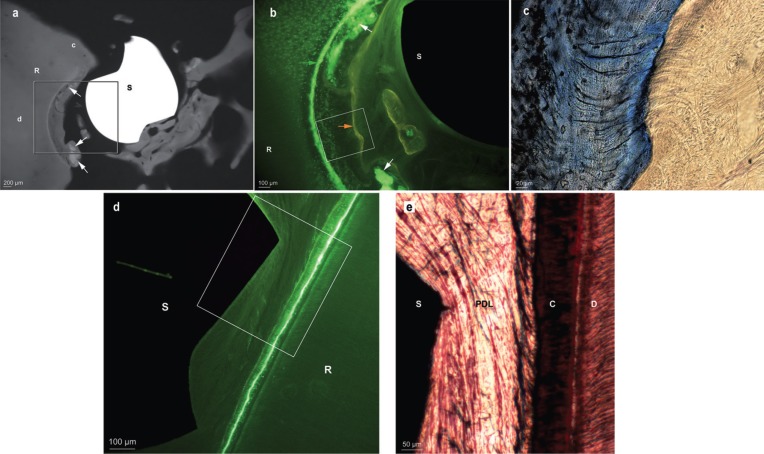
A, Microradiograph showing screw 3 (29 weeks) damaging the root at the apex level, and showing hypermineralized bone debris (white arrows) included in dental reparative tissue. Some mineralized tissue was also present on the side opposite to the root, and was probably contributing to screw stability. B, Enlargement of framed area in Fig 3a, under UV light, showing cementum apposition between calcein green (green arrow) and alizarin complexone (orange arrow) labelling. C, Enlargement of the framed area in Fig 3b, observed in the stained section. Fibres connecting the tooth and the bony fragments around the screw are clearly visible. S: screw - R: root - c: cementum - d: dentin. D, Section examined under fluorescent light, showing screw 6 placed into the PDL, but generating no root lesion as demonstrated by the continuous calcein green labelling. E, Enlargement of the framed area in Fig 3d, observed in the stained section. R: root - S: screw - C: cementum - D: dentin.

By contrast, in the dogs euthanatized after 29 weeks (screws 3, 4 and 5), new, radiopaque cementum partly filled the root defect and was labelled by calcein, on the inner side, and alizarin, on the external side (Fig. 3a,b). Around those screws, hypermineralized tissue fragments produced during drilling and diffusely labelled by calcein were nearly completely enclosed in new cementum. In the stained sections, fibres connecting the tooth and the bony fragments around the screw were clearly visible. Similar to the PDL fibres, they were anchored in the new cement. No signs of inflammation were observed either in the pulp or near the cement (Fig. 3c).

Screw 6, placed into the PDL but not in direct contact with the root, did not produce any root resorption, as confirmed by the uninterrupted calcein labelling (Fig. 3d). No inflammation was observed at the PDL or at the pulp level. Periodontal fibres were surrounding the screw without any sign of pathology (Fig. 3e).

Importantly, no signs of root resorption were observed around any of the root lesions observed.

No correlation was observed between the presence of tissue repair and screw stability or loading status.

## Discussion

Skeletal anchorage devices, such as miniplates, need to get a better insight into their side-effects. Root damage is a frequent, somewhat underestimated complication. This sample was part of a larger experiment (10), in which the frequency of root damage concerned 5 out of 40 maxillary miniplates (12.5%) and 6 out of 80 anchoring screws (7.5%). However, the damage systematically concerned the maxillary canine which is very long in dogs, and could therefore not be representative of clinical situations. However, in clinical conditions, Kim and coworkers reported that 7 out of 31 maxillary miniplates (22.6%) and 7 out of 74 anchoring screws (9.5%) damaged a root (11). In that study, the high frequency of root damage was always due to the most proximal screw of the miniplate, which could be related to the very short connection arm of the miniplate model used (C-tube, KLS Martin), obviously bringing the most proximal screw closer to the dental roots. If root damage due to orthodontic miniplates with longer connection arms can be expected to be slightly less frequent than in that report, still root damage has to be considered with care since no other frequency is reported so far in the literature.

Indeed, although limited root trauma has been shown to be repaired by cementum apposition, extensive lesions might not necessarily heal so uneventfully. Several authors observed cementum repair within 6 to 12 weeks after moderate root damage due to miniscrew placement (3-5,7,9,12-15), even if the screw was left in contact with the root (16,17). Repair involved deposition of cellular cementum on the root surface, increasing with time (12), with regeneration of the PDL (4,6,7). On the other hand, other authors observed little or no repair when the miniscrew was left in contact with the root, suggesting that relief from pressure is needed to initiate cementum deposition (18).

Up to now, repair after miniplate-related damage has not been reported. The healing reactions observed around screws 3, 4 and 5 in the present study, with root damage limited to the cementum or dentin, were coherent with the repair process previously described for limited trauma due to miniscrews. In addition, no periodontal or pulpal inflammation was observed.

However, if most authors claim that root lesions are well repaired, recent findings warn orthodontists that abnormal healing is the consequence of extensive damage involving inflammatory infiltrate or pulpal invasion, with no improvement over time (12,16,17). By contrast, Dao and coworkers reported no pulpal necrosis after penetration of the pulp by miniscrews after 12 weeks of follow-up, but observed tooth ankylosis which is also a severe complication, especially when orthodontic movement is the aim (9,13).

In the present study, absence of healing as well as lack of new mineralized tissue were observed after 7 weeks, although inflammatory signs were not present.

Although new cementum was covering the defects after 29 weeks, the small sample size and the reduced aspect of these lesions compared to the 7-weeks group screws do not allow to conclude that 7 weeks represent a too short period to initiate cementum healing. Nevertheless, it can be stated that limited damage shows repair after 29 weeks, and that healing of extensive lesions is hazardous. Although this has to be verified on a larger sample, contact of the screw with the PDL did not induce root resorption. Similarly, root damage was not followed by root resorption, suggesting no aggravation of the initial lesions due to the repair process. In contrast to this finding, Kim & Kim observed signs of root resorption even when the miniscrew was just in proximity to the root, suggesting a role of the pressure over the PDL created by the miniscrew (18).

The severity of root damage raises the question of the necessity of a drilling procedure before screw insertion. The present study attested the difficulty even for an experienced surgeon to discriminate between osseous and dental tissue during the drilling procedure. This might argue in favor of a non-drilling procedure, as placement torque of self-drilling screws is twice as high when root contact is present than without (12). However, if increased resistance should be used as an indicator of possible root contact during miniscrew placement (7), other investigators reported to feel no obvious change in resistance when contacting the cementum, even when fracturing the root while inserting a self-tapping screw (19). According to a systematic review, the insertion torque of miniscrew contacting a root is always higher than the insertion torque of miniscrew inserted into the bone, therefore an insertion procedure involving the use of an handpiece and continuous real-time control of the insertion torque could be suggested (20).

The small sample size does not allow making correlations between root damage and screw loading or screw stability. Concerning miniscrews, root contact has been reported to be a major risk factor for miniscrew failure (6,19,21) while other authors reported that minimally damaged dental roots did not adversely affect the healing process (7,9). Only one group investigated root contact with Cone Beam CT after miniplates placement in patients and concluded that root penetration had minimal effects on the successful stabilization of miniplates (11). The present experiment, though conducted on animals, showed a similar tendency, as 4 out of 6 screws remained stable throughout the experiment.

Despite the limitation of a small sample size, the results of the present study can be helpful to improve our knowledge on root damage repair, since the damages from miniplates insertion were never assessed before, and a systematic review on the topic reported only two studies at low risk of bias (14). In addition, in such particular research topic, animal studies provide precious information, since in human studies where teeth are extracted the assessment of periodontal attachment is difficult and it is not possible to evaluate the presence of ankylosis (16).

## Conclusions

Minimal root damage has shown potential to heal: apposition of new cementum was seen in case of limited root damage, with a thick layer of mineralized tissue observed on the lesions. By contrast, extensive root damage was not followed by any repair process: healing of extensive lesions is problematic. Precise position of insertion of the miniplates is thus of utmost importance.
